# Boundary layer wind tunnel measurement datasets of airflow study in an asymmetric naturally ventilated livestock building

**DOI:** 10.1016/j.dib.2026.112592

**Published:** 2026-02-14

**Authors:** Long Chen, Lars Thormann, Thomas Amon, Qianying Yi, David Janke

**Affiliations:** a Department of Sensor and Modelling, Leibniz Institute for Agricultural Engineering and Bioeconomy, Potsdam, Germany; b Institute for Animal Hygiene and Environmental Health (ITU), Free University Berlin, Germany

**Keywords:** Atmospheric boundary layer, 2D velocity, LDA, Wind profile, CFD validation

## Abstract

This data article presents high-resolution airflow and turbulence measurements obtained in and around a 1:50 scaled asymmetric dairy barn model using a boundary layer wind tunnel. The datasets are organized into two categories: (1) measurement data of boundary layer profile in the empty wind tunnel to assess flow stability, Reynolds number independence, and characterize vertical wind profile, and (2) airflow measurements at a cross-sectional plane inside and outside of the scaled model under the established boundary layer, comprising a total of 233 measurement positions. All air velocity measurements were acquired using a two-dimensional Laser Doppler Anemometry (LDA) system to ensure a sufficiently long sampling duration for robust statistical analysis. The datasets are primarily designed for validation and refinement of computational fluid dynamics (CFD) models as an essential step prior to following CFD applications. A validated CFD model can then be employed to estimate airflow and emission. Therefore, those datasets provide pivotal references for numerical modelling, and are valuable for further investigations of wind-driven ventilation performance as well as emission characterization in naturally-ventilated livestock buildings.

Specifications Table

**Table:** 

Subject	Earth & Environmental Sciences
Specific subject area	Physical Modelling, Natural Ventilation, Airflow Characterization
Type of data	Table, Image, Figure, 3D drawing. Raw, Analyzed, Processed.
Data collection	A boundary-layer wind tunnel (3 m x 2 m cross-section, 19.5 m flow section); inlet freestream velocities were measured by a Prandtl tube connected to a pressure transducer MKS Baratron Type 120A (MKS Instruments, Andover, MA, USA); 2D air velocity and turbulence were measured by the Laser Doppler Anemometry (LDA) from Dantec (Dantec Dynamics, Skovlunde, Denmark) combined with the BSA Flow Software package; a fog generator Tour Hazer II (Smoke Factory, Burgwedel, Germany) was employed to produce seeding particles for LDA measurements; temperature, relative humidity, and static pressure of ambient air were recorded by a FHAD 46x sensor with ALMEMO D6 plug (AHLBORN Mess- und Regelungstechnik GmbH, Ilmenau, Germany).
Data source location	Institution: Leibniz Institute for Agricultural Engineering and Bioeconomy (ATB) City/Town/Region: Potsdam, Brandenburg Country: Germany
Data accessibility	Repository name: Mendeley Data Data identification number: doi:10.17632/c4kt7xn2b5.3 Direct URL to data: https://data.mendeley.com/datasets/c4kt7xn2b5/3
Related research article	

## 1 Value of the Data

• Our study provides high-resolution wind tunnel measurements of airflows in and around an asymmetric, naturally ventilated livestock building model (geometric model provided), which benefits researchers in livestock housing design for evaluating and improve ventilation strategies by providing realistic validation datasets for complex geometries. Because they are urgently needed to validate real-world simulations targeting beyond the standard benchmark cases, such as lid-driven cavity or backward-facing step [[1], [2], [3], [4], [5]].
• The velocity datasets offer a valuable benchmark for validating numerical simulations, particularly for CFD models used in the study of livestock ventilation systems [1,6,7]. The spatially resolved measurement points allow for detailed comparisons between experimental and simulated results, supporting robust model calibration and validation efforts.
• The pointwise instantaneous air velocity data present velocity fluctuations that is ideal for comparing time-series turbulence statistics and transient structures without interferences. Therefore, the published data facilitate researchers to reproduce the boundary conditions and validate transient large eddies simulations (LES) [8,9].
• The datasets can be employed as references in indoor air quality and emission research, such as assessing ventilation efficacy and studying contaminant transport [[10], [11], [12]]. Researchers can reuse the dataset for training surrogate models and conducting uncertainty or sensitivity analyses related to airflow modelling and ventilation performance under varying boundary conditions.
• The datasets may also serve as a reference for experimental planning and sensor placement optimization in scaled or in-situ airflow studies, enhancing the design of future measurement campaigns under similar conditions [11,13].

## 2 Background

Validating CFD models is essentially required before any application of simulations. Therefore, a high quality, well-organized and representative validation dataset is always demanded for investigations using numerical models. However, field measurements are easily interfered by different factors and are hard to ensure the quality of data. Therefore, a fully controlled experiment environment is highly needed. A large wind tunnel facility at the Leibniz Institute for Agricultural Engineering and Bioeconomy (ATB), Germany, was deployed to create an atmospheric boundary layer to facilitate air velocity measurements. The boundary layer wind tunnel (BLWT) has a total length of 28.5 m in the streamwise direction and a cross-section of 3 m (width) × 2 m (height). By constructing scaled model and desired boundary profile, we are able to measure airflow in a systematic manner. Particularly, in the field of agricultural environment, wind tunnel experiments can provide considerable and reliable airflow measurement data for natural ventilation as well as for emission studies.

## 3 Data Description

In total, four Excel files (processed data), four compressed archive files (raw data), and one geometry data of the study barn are presented. For the definition of specific measurement lines, please refer to Section 3.

**Fig. 1:**
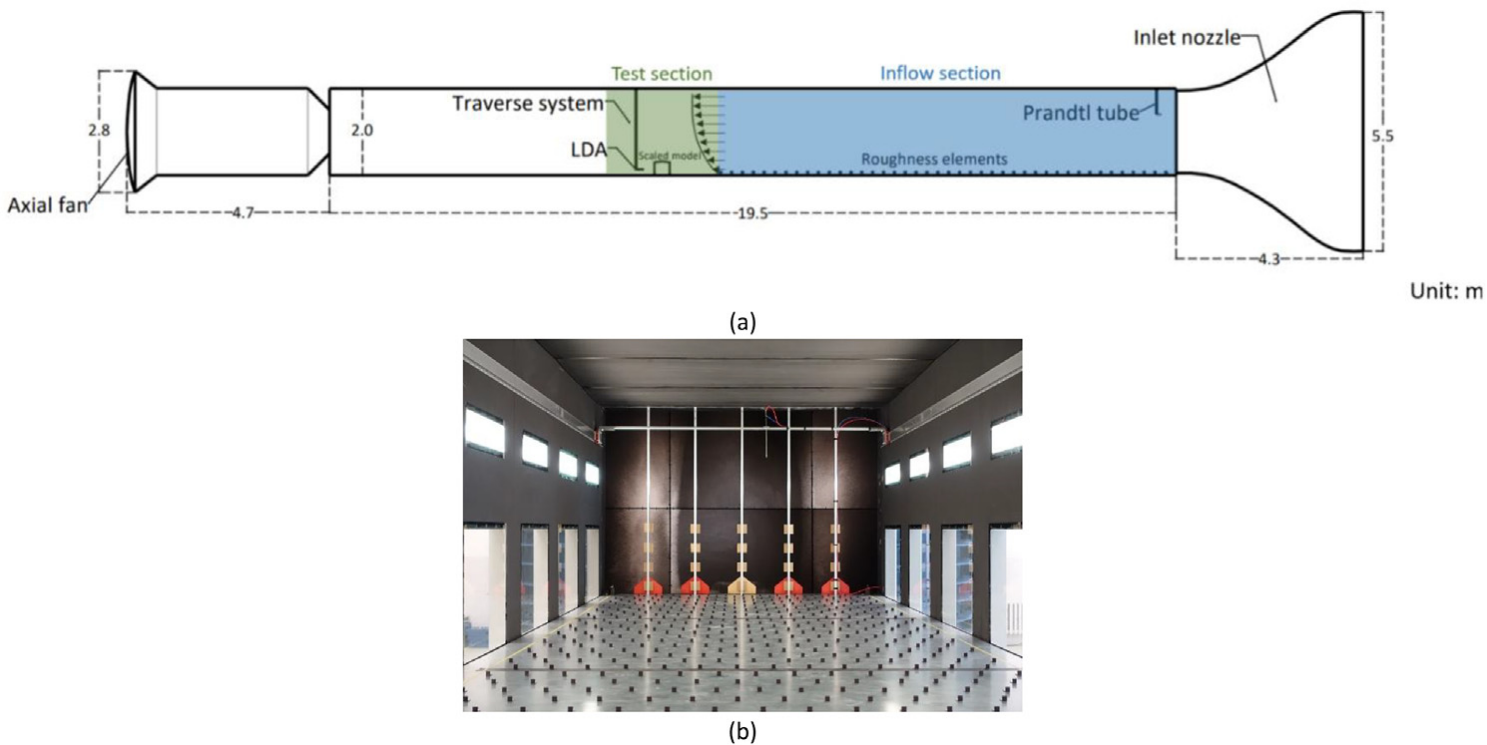
Layout of wind tunnel experiment. (a) A schematic sketch of the boundary layer wind tunnel; (b) spires at the inlet and roughness elements of the inflow section.
(3) Wind profile properties and repeatability study (Wind_profile_analysis.xlsx)
  This Excel file contains a spreadsheet of wind profile measurements at the center location of inflow (“SCL0”) with three replicates. The spreadsheet also includes graphs of temporally averaged streamwise and vertical velocity profiles at increasing heights (15 – 600 mm). A wind profile for streamwise velocity is calculated and presented based on a power-law equation in the spreadsheet. To quantify the variability between three repeats, we calculated coefficient of variance (CV) for u and w respectively and compared the mean value at each measurement position.
(4) CFD validation study (Validation_Dataset_GK-575.xlsx)
  The Excel file has three spreadsheets: one is the coordinates for 11 validation lines combined with the illustration in Fig. 4; the other two sheets (“u_profile” and “w_profile”) contain temporally averaged streamwise and vertical velocities at every single measured location.
(5) Datasets of velocity component for streamwise velocity profile consistency assessment (Raw_streamwise-velocity-consistency_assessment.zip)
  This compressed archive file contains raw measurement data at the 6 vertical lines (20 points on each line) for the stability study that was exported directly from BSA software, including temporal streamwise and vertical velocity components captured by LDA.
(6) Two-dimensional datasets of velocity component for Reynolds number independence study (Raw_*Re*-Independence_study.zip)
  Similarly, this compressed archive file contains raw measurement data at the same vertical line (“SCL0”) with five different freestream inlet velocity (4, 6, 8, 10, 12 m/s).
(7) Two-dimensional datasets of velocity component for wind profile properties study (Raw_Wind-profile-properties_study.zip)
  This compressed archive file contains raw measurement data at the same vertical line (“SCL0”) in three replicated measurements with the freestream inlet velocity of 8 m/s.
(8) Two-dimensional datasets of velocity component for CFD validation study by LDA (Raw_validation_ds_GK-575.zip)
  This compressed archive file contains raw measurement data for the entire 233 points for validation study exported directly from BSA software, including temporal streamwise and vertical velocity components captured by LDA.
(9) Three-dimensional geometry of the study barn model (GK_geom_v3_1–50scale_with_dimensions.STL)

(1) Consistency assessment of streamwise velocity profiles (Streamwise velocity_consistency_assessment.xlsx)
  The Excel file contains the list of temporally averaged streamwise velocity component and standard deviation at six vertical lines designed for consistency assessment (Fig. 3). Each line includes 20 measurement positions. A graph of showing the comparisons between measurements at the six lines is included in the spreadsheet, which depicts the relationship between normalized height and normalized streamwise velocity.
(2) Reynolds number independence study (Re_Independence_analysis.xlsx)
  The Excel file presents analysis of streamwise and vertical velocity profiles at the line “SCL0” (Fig. 3) with 5 different freestream wind speed ($u_{inlet}$) at the wind tunnel inlet (Fig. 1(b)). The graphs of the comparisons of u and w under each $u_{inlet}$ are also included in the spreadsheet. In addition, parameters required to calculate building Reynolds number ($Re_{B}$) are shown in the spreadsheet with the calculated $Re_{B}$ value presented.

This .STL file consists of the geometry of 1:50 scaled dairy barn model with annotations of key dimensions, which can be used for further simulations and data reproduction.

## 4 Experimental Design, Materials and Methods

### 4.1 Wind tunnel experimental design

The boundary layer wind tunnel (BLWT) consists of an air inlet equipped with honeycombs, an air outlet fitted with an axial fan, and a 19.5 m-long flow section (Fig. 1(a)). At the entrance of the inflow section, five vertical obstacle lines were installed, each formed by four vertically aligned square spires (0.1 *m* × 0.1 m), with the bottom spire overlapped by a pentagon-shaped spire (Fig. 1(b)) to create desired turbulence [14]. Downstream of the spires, 42 rows of roughness elements were installed to develop the desired boundary layer. A single size of roughness element (0.02 *m* × 0.02 m) was employed and arrayed in staggered with a 0.25 m spacing between rows, covering a total length of 10.50 m. The spires and roughness elements together ensure the inflow present a grassland level turbulence behavior at 1:50 scale approaching the test section.

**Fig. 2:**
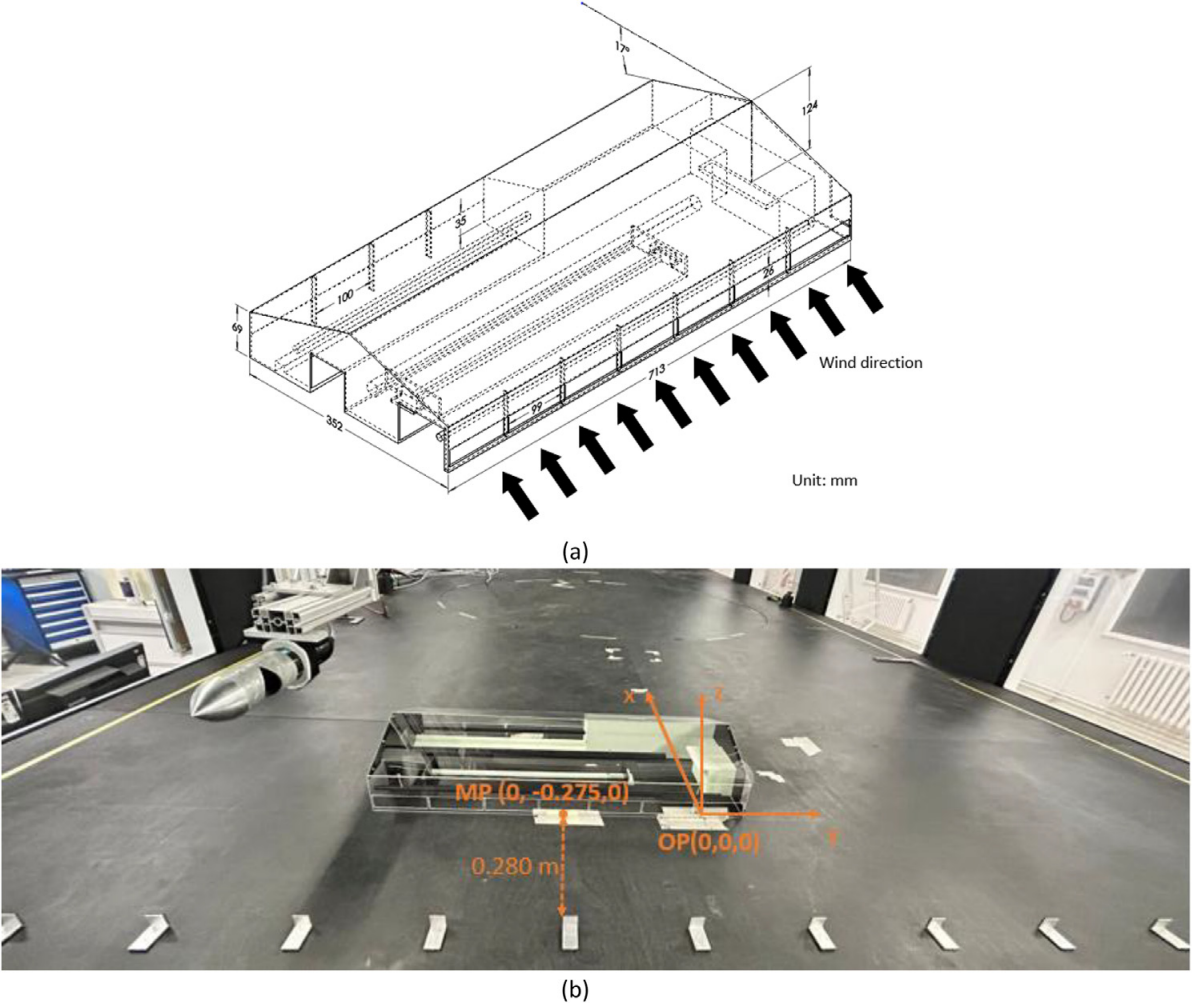
A 1:50 scaled dairy barn model. (a) Dimensions of the scaled model with indication of inflow direction (cross wind); (b) positioning of the scaled model at wind tunnel: 0.28 m downstream the last row of roughness elements, with illustrations of original point (OP) and the midpoint (MP) of upwind sidewall bottom edge.

A scaled model of a naturally ventilated dairy barn was employed in this study (Fig. 2). The prototype barn, located in the state of Brandenburg Germany, was designed to house approximately 60 cows. The scaled model represented a 1:50 geometric reduction of the full-scale barn and was constructed from 2mm-thick acrylic glass supported by rigid frames (Fig. 2(b)). The model features an asymmetric configuration, characterized by two distinct sidewall, along with door openings at both gable ends. The interior includes two elevated bedding areas and additional minor ground-level features. Three ventilation ducts for provision of fresh air to the animals were integrated into the model above the bedding area. Dimensions of the scaled model are presented in Fig. 2(a). The scaled dairy barn model was positioned within a stable turbulence zone based on preliminary test of boundary profile where its upwind sidewall aligned to the x-axis and centered at (x = 0 mm, y = −275 mm) and is located 0.28 m downstream of the last row of roughness elements (Fig. 2(b)). The approaching wind is perpendicular to the side with 7 identical (99×26 mm) openings (Fig. 2(a)). Its blockage ratio was 0.7 %, well below the 5 % limit recommended by VDI 3783/12, allowing tunnel effects to be neglected [15].

**Fig. 3:**
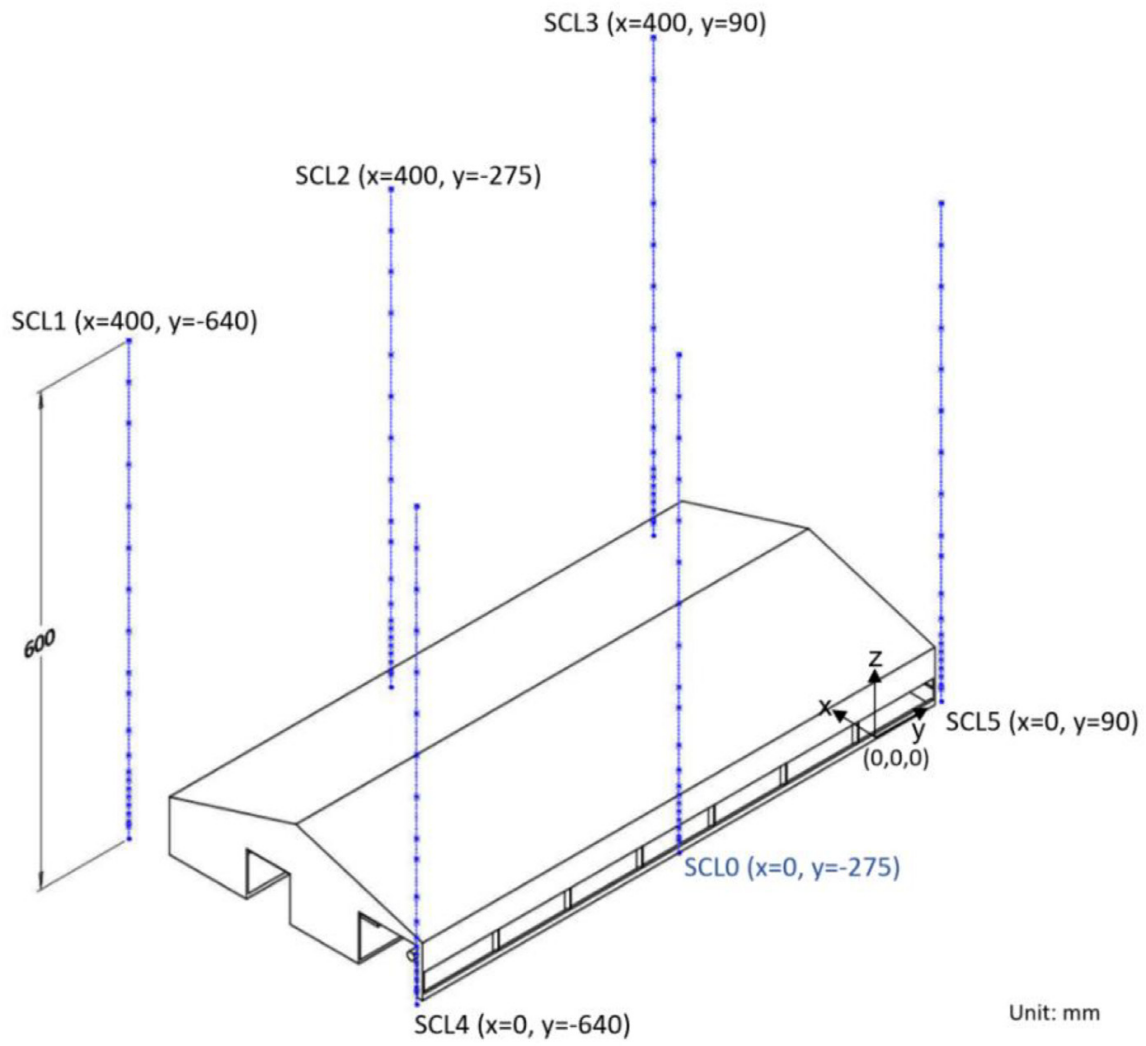
Consistency assessment of streamwise velocity profiles. An illustration of six vertical lines (0.6 m high) with 20 measurement positions at each line. The corresponding coordinates for individual lines and the origin are annotated. Note the measurement was conducted without scaled model.

To establish a stable boundary-layer airflow representative of farmland terrain, wind profiles in the region of interest were measured. Six vertical measurement lines, each containing positions from z= 0.015 m to z= 0.6 m in height (SL0 to SL5), were selected to compare their wind profiles at an inlet velocity $U_{inlet}=$ 8 m/s (Fig. 3). The height and velocity are normalized by H and $u_{H}$, where H= 130 mm as the model height and $u_{H}$is the mean streamwise velocity component at the model height of the approaching flow at each vertical line.

To ensure a fully developed turbulent flow in the test section, a Reynolds number independence study of the approaching flow was conducted. Wind profiles were measured at Line “SCL0” (Fig. 3) with 20 vertical measurement positions. The profiles were compared at inlet velocities of 4, 6, 8, 10, and 12 m/s. Based on the calculated bulk Reynolds number$\;Re_{B}=$ 64,349 and $U_{inlet}=$ 8 m/s was selected for all subsequent experiments since $R_{eB}$ is greater than the critical value of 4000 [16,17]. A Repeatability study was also performed at Line “SCL0” by repeating the measurements three times. The power-law exponent was then derived from these measurements.

**Fig. 4:**
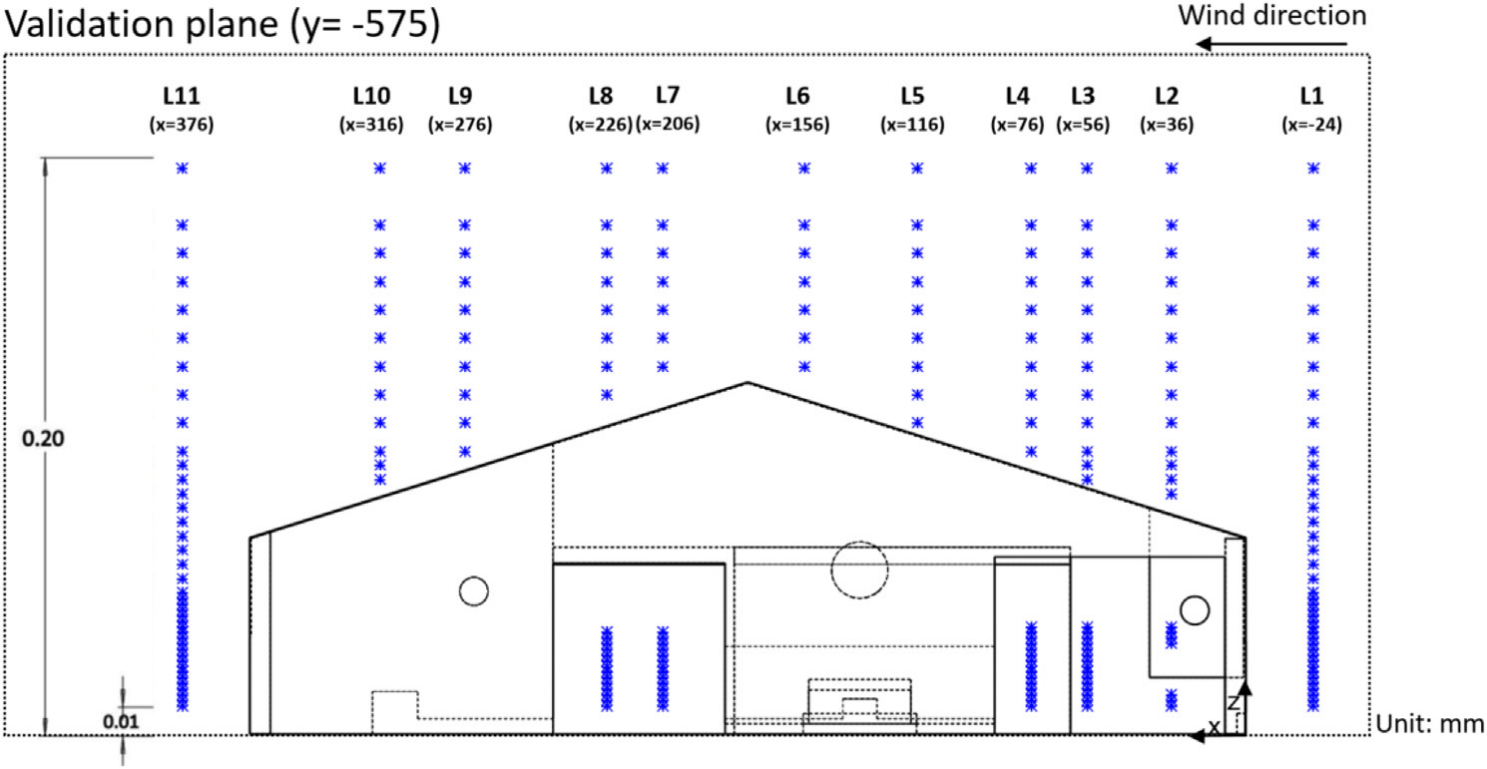
Validation plane including 233 measurement positions at 11 vertical lines. Blue dots represent individual measurement positions. The lowest measurement is z= 10 mm and the highest is z= 200 mm.

For the validation study, eleven vertical measurement lines were defined at y = −575 mm, ranging from z= 0.015 m to z= 0.6 m, covering regions both inside and outside the barn (Fig. 4). A total of 233 measurement positions were acquired using Laser Doppler Anemometry (LDA) with high data quality. The model was only installed for the validation study; boundary-layer development, Reynolds independence, and wind profile measurements were performed without the model present.

### 4.2 Instrumentation

Ambient temperature, relative humidity, and static pressure were recorded hourly using an FHAD 46x sensor with an ALMEMO D6 plug (AHLBORN Mess- und Regelungstechnik GmbH, Ilmenau, Germany) to determine temporal air density and adjust the wind speed at inlet ($U_{inlet}$) accordingly, which was then measured using a Prandtl tube connected to an MKS Baratron Type 120A pressure transducer (MKS Instruments, Andover, USA) positioned at the center of the inflow section entrance, 1.42 m above the tunnel floor.

Air velocity and turbulence in and around the scaled model were measured using a 2D fiber-optic LDA equipped with a 300 mW Modu-Laser (Stellar-PRL ML/300) manufactured by Dantec Dynamics (Skovlunde, Denmark) operated with the BSA Flow Software package v7.50 (Dantec Dynamics) on a Windows 10 operating system. The LDA probe, 0.06 m in diameter and 0.45 m in length with a focal length of 0.25 m, was mounted on a three-dimensional computer-controlled traverse system, enabling automated probe positioning with an uncertainty of <0.1 mm. The probe was inclined at approximately 23° to simultaneously measure streamwise and vertical velocity components. For seeding, a Tour Hazer II fog generator (Smoke Factory, Burgwedel, Germany) was placed at the tunnel inlet to generate tracer particles for the LDA measurements using a designated tour hazer fluid, producing aerosol particles with a typical aerodynamic diameter in the range of approximately 0.5 – 1.0 µm, suitable for LDA measurements.

### 4.3 Data acquisition

The streamwise (u) and vertical (w) velocity components were measured using the 2D LDA. The measurements at each position were captured continuously until either the sampling number reached 60,000 readings or the maximum sampling time reached 300 s before moving to the next measurement position. A 10 s pause between two consecutive measurements was set in order to minimize the disturbance of the movement of the LDA probe to the flow field.

### 4.4 Data calculation

Raw data is firstly processed by calculating the mean, variances, and covariance from time series data collected by LDA in a sampling period time T (s) as follows:

(1) $$\bar{u}=\frac{1}{T}\cdot \int_{0}^{T}u\left(t\right)dt$$

(2) $$\bar{w}=\frac{1}{T}\cdot \int_{0}^{T}w\left(t\right)dt$$

(3) $$\sigma_{u}^{2}=\frac{1}{T}\cdot \int_{0}^{T}{\left(u\left(t\right)-\bar{u}\right)}^{2}dt$$

(4) $$\sigma_{w}^{2}=\frac{1}{T}\cdot \int_{0}^{T}{\left(w\left(t\right)-\bar{w}\right)}^{2}dt$$

(5) $$\bar{u^{\prime }}\bar{w^{\prime }}=\frac{1}{T}\cdot \int_{0}^{T}\left(u\left(t\right)-\bar{u}\right)\left(w\left(t\right)-\bar{w}\right)dt$$

To calculate $R_{eB}$ the following equation is employed [17]:

(6) $$R_{eB}=\frac{u_{H}\cdot D}{v}$$

(7) $$D=\frac{2W_{M}\cdot H_{M}}{W_{M}+H_{M}}$$

where $W_{M}$ is model width, $H_{M}$ is model height, D represents characteristic length, v is kinetic viscosity of air, and$\;u_{H}$ stands for streamwise velocity at model height. Theoretically wind profile generated in the wind tunnel should be conformed to a mathematical relationship for the vertical extrapolation of free stream wind speed in the atmospheric surface layer. Thus, the derived vertical profile of the time-averaged velocity for the near-ground boundary layer wind profile is described as following [15]:

(8) $$\bar{u}\left(z\right)=\frac{u^{*}}{k}\cdot \ln\frac{z-d_{0}}{z_{0}}$$

where $\bar{u}\left(z\right)$ (m/s) stands for streamwise mean velocity component at a height z (m); z is the height from ground; $z_{0}$ (m) is the full-scale roughness scale; $d_{0}$ (m) is zero-plane displacement height that is considered negligible for neutral atmospheric conditions; $u^{*}$ (m/s) is wall friction velocity that is used to characterize the turbulent momentum exchange near the ground; k represents the von Karman constant that is assigned as 0.4 hereby. In practical, the vertical streamwise mean velocity profile can be fit to a power-law equation based on actual measurements using following equation:

(9) $$\bar{u}\left(z\right)=u_{ref}\cdot {\left(z\;/\;z_{ref}\right)}^{\alpha }$$

where $u_{ref}$ (m/s) is the mean streamwise air velocity at a reference height $z_{ref}$ (m) [15]. In this study, we presented the calculation of α in the dataset of Wind_profile_analysis.xlsx.

By knowing $z_{0}$ and $u^{*}$, the roughness Reynolds number$\;Re_{z_{0}}$can be calculated using the following equation:

(10) $$Re_{z_{0}}=u^{*}\cdot \frac{z_{0}}{v}$$

In this study, we characterized the wind profile in a power-law equation with α= 0.17 and $z_{0}=$ 0.014 m (full scale), which fell in the range representing a moderate rough terrain [15,18,19]. Additionally, we calculated $R_{eB}$ and $R_{ez_{0}}$ as 64,349 and 6.61, both are greater than the critical value 4000 and 2.5 [16], respectively. All these critical parameters fall within the recommended range as required as criterion for evaluating the quality of wind tunnel experiment.

### 4.5 Normalization and repeatability

To check the stability of boundary layer at multiple positions, we calculate the normalized streamwise velocity component for u and the normalized height using $u_{H}$ and H for comparison. To test the wind profile’s repeatability, we triplicated the measurement at the same position to characterize the approaching flow’s velocity profile [20]. We used coefficient of variance (CV) to quantify the variability (Fig. 3). All the calculations are appended in the Wind_profile_analysis.xlsx.

## Limitations

To keep the consistency, velocity components are 2D through the entire study, u and w, with no lateral components measured. Besides, the number of velocity measurement inside the model was limited due to the blockage of opaque structures of the model, which resulted in extremely low data rates at some positions. For the measurements close to the ground, we observed the vertical velocity always showed a very low magnitude, which resulted in larger variations during the statistical analysis.

## Ethics Statement

The authors have read and follow the ethical requirements for publication in Data in Brief and confirmed that the current work does not involve human subjects, animal experiments, or any data collected from social media platforms.

## CRediT Author Statement

**L. Chen:** Conceptualization, Investigation, Formal analysis, Writing – original draft, Writing – review & editing. **L. Thormann:** Conceptualization, Software, Investigation, Formal analysis, Writing – review & editing. **T. Amon:** Conceptualization, Funding acquisition, Writing – review & editing, Supervision. **Q. Yi:** Formal analysis, Investigation, Writing – review & editing. **D. Janke:** Conceptualization, Investigation, Funding acquisition, Writing – review & editing, Supervision.

## Data Availability

Mendeley DataVelocity datasets of an asymmetric dairy barn model by LDA in BLWT (Original data).
